# Editorial: Full and Partial Hospitalization Interventions for Eating Disorders

**DOI:** 10.3389/fpsyt.2021.775715

**Published:** 2021-10-13

**Authors:** Enrica Marzola, Renee D. Rienecke, Valentina Cardi, Cheri A. Levinson

**Affiliations:** ^1^Department of Neuroscience “Rita Levi Montalcini,” Eating Disorders Center, University of Turin, Turin, Italy; ^2^Eating Recovery Center/Pathlight Mood and Anxiety Centers, Chicago, IL, United States; ^3^Department of Psychiatry and Behavioral Neuroscience, Northwestern University, Chicago, IL, United States; ^4^Department of General Psychology, University of Padova and Department of Psychological Medicine, King's College London, London, United Kingdom; ^5^Department of Psychological and Brain Sciences, University of Louisville, Louisville, KY, United States

**Keywords:** anorexia nervosa, bulimia nervosa, adolescents, hospitalization, partial hospitalization, residential, avoidant/restrictive food intake disorder, adults (MeSH)

Eating disorders (EDs) are complex psychiatric illnesses posing a severe burden on patients and their significant others. Physical and psychological sequelae can occur, with low quality of life and high mortality rates complicating this picture even further. Treatment is challenging and only 50% of patients respond to gold-standard treatments. There are many psychological hallmarks leading to treatment resistance (1), and state-related pernicious (sometimes life-threatening) conditions (2). In this light, for a substantial number of patients, an intensification of outpatient treatment is needed over the course of illness. For example, from 1999 to 2009, hospitalizations of patients affected by EDs increased for all age groups (3), and during the COVID-19 pandemic hospitalisations increased in particular among the youngest (4). Therefore, full and partial hospitalization interventions become necessary for a substantial portion of patients. Although it has been stated that recovery from EDs may entail a long journey [“hope despite mortal risk” (5) p. 1309], it is also true that an ongoing ED tends to exacerbate patients' depression, anxiety, and chronic stress (6), thus generating a slippery slope toward unfavorable outcomes. Further, intensive treatments are costly; the average cost of inpatient ED treatment is $2,267 per day, residential/partial-hospital are $1,000 per day, not accounting for the high costs associated with acute hospitalization and medical stabilization (7, 8). Despite the high need and cost of intensive treatments, there is scant research on the effectiveness and clinical utility of these approaches. Therefore, this call for research aimed to stimulate scientific debate on treatment of one of the most common types and difficult phases of an ED. Our ultimate goal is to begin to improve the state of science behind intensive treatments for EDs.

It is noteworthy that one-third of the contributions to this collection focused on adolescents with EDs. Interestingly, Baudinet and Simic conducted a comprehensive review of 49 studies of day programs for adolescents with mixed EDs finding substantial evidence of their effectiveness when compared to inpatient treatment. In line with this, Zanna et al., after retrospectively comparing adolescents who followed inpatient treatment and adolescents who received partial hospitalization, provided further support for day programs as effective alternatives to hospitalization for young patients with anorexia nervosa (AN). Relatedly, Litmanovich-Cohen et al., found day programs to be an effective strategy for former adolescent inpatients with EDs. In fact, those who completed a post-hospitalization day program reported greater improvement at follow-up when compared to adolescents who did not undergo such an intervention. Finally, three studies provided data on the broad spectrum of family-based interventions. Datta et al. raised the question as to whether hospitalization could impact weight restoration for adolescents undergoing outpatient treatments for AN, finding a different impact depending on the baseline treatment provided. That is, those undergoing Adolescent Focused Therapy gained more weight during hospitalization than those receiving Systemic Family Therapy and Family-Based Treatment. Mensi et al. highlighted the relevance of including family members when working with adolescents with a restrictive form of EDs including restrictive and binge-purging subtypes of AN, avoidant/restrictive food intake disorder (ARFID), atypical AN, other specified feeding or EDs with restrictive characteristics. Finally, Wallin and Holmer focused on the Family Treatment Apartment (FTA) model, which was pioneered as an alternative to psychiatric inpatient care. Comparing short- and long-term outcomes of adolescents with AN receiving FTA vs. inpatient stay, the authors found favorable outcomes for those who underwent FTA with respect to readmissions due to weight loss, general psychiatric pathology and quality of life. Overall, these studies provide evidence for the efficacy of intensive treatments for adolescents with EDs, and describe which specific treatment modalities within intensive treatment might be most effective. More and similar research is needed in adult populations also given the lack of evidence-based effective approaches available for adults with EDs (9).

Several contributions focused on adults with EDs; of those, only one paper investigated a day program intervention. Tenconi et al. analyzed the impact of undergoing a partial hospitalization treatment on cognitive functioning in adult patients with AN. They found that decision-making improved after treatment, while cognitive monitoring and cognitive inhibition remained stable over time. While Body Mass Index (BMI) and duration of illness predicted treatment response, neuropsychological performance did not contribute to the prediction model.

In addition to the aforementioned study, several papers of this Research Topic focused on inpatients. It is of relevance that treating patients who are in an acute phase of their ED can stimulate the pioneering testing of novel interventions. In many situations, patients are hospitalized without motivation to address ED behaviors, in part because of the intrinsic ego-syntonic (i.e., overall acceptable to the patients) nature of the ED. As a result, many patients are placed in a therapeutic setting without being ready to start the recovery process. In this light, Ziser et al. explored a novel intervention called “Motivation-Enhancing Psychotherapy for inpatients With Anorexia Nervosa (MANNA)” aimed to enhance readiness for behavioral change. The authors conducted a randomized controlled trial (RCT) evaluating the feasibility of a novel 10-week program for individual psychotherapy sessions using elements of motivational interviewing. These preliminary data pointed to a better outcome, in terms of hospitalization completion, for those patients with AN who received MANNA. Echoing this line of research, it is noteworthy that Smith and Tchanturia investigated the use of “huddles” (sometimes referred to as treatment teams), namely time-limited and focused meetings, to support clinical teams. These findings are of high interest because of the significant and often unaddressed burden posed by the disorder on clinicians involved in the treatment of EDs. The authors found that huddles were rated as highly useful and could have potential in higher-level of care for EDs. Thompson-Brenner et al. analyzed the long-term effects of a transdiagnostic, evidence-based treatment for patients with mixed EDs requiring a residential level of care, named “Renfrew Unified Treatment of Eating Disorders and Comorbidity,” and found positive outcomes for those who underwent this intervention. Outcome measures included eating, depressive, and anxiety symptoms. Interestingly, those who responded less well to the intervention had reported marked levels of emotional dysregulation. Outcomes were maintained over a 5-year timeframe, providing substantial support for this type of intervention during residential care. Finally, addressing the additional challenges brought about by the COVID-19 outbreak, Latzer et al. analyzed the pros and cons of a virtual home-based treatment during the COVID-19 pandemic for Ultra-Orthodox young women previously hospitalized because of an ED. The authors reported that online therapy was effective for patients and parents motivated to undertake virtual treatments (e.g., parents using their COVID-19-related presence at home to further assist their children during meals); in contrast, virtual home-based treatment hindered treatment under specific circumstances (e.g., lack of suitable online devices, over-crowded families, specific religious beliefs). We are encouraged by the burgeoning literature on novel interventions in higher levels of care and look forward to seeing the field continue to progress in this area.

Four papers focused on treatment predictors of severe EDs. Redgrave et al. conducted a study on inpatients diagnosed with severe and enduring AN, investigating weight restoration as a predictor of follow-up clinical status. They found that those with greater weight restoration showed significantly better outcomes at 6-month follow-up. Simpson et al. investigated the predictors of full hospitalization or residential treatment after receiving day treatment for patients with mixed ED diagnoses. Low BMI, residential treatments in the past, and anxious and depressive comorbidity were found as relevant predictors of needing a higher level of care after partial hospitalization. Also, Kaufmann et al. highlighted the role of BMI as an outcome predictor in AN. Not only low lifetime BMI predicted weight at the admission of inpatient treatment, but higher lifetime BMI also predicted a positive outcome both at discharge and at follow-up. Finally, Marzola et al. investigated the phenomenon of readmission and “revolving door” (i.e., rapid readmission) in AN. Focusing on predictors of time-to-readmission, the authors found drive for thinness as a robust predictor of a shorter time to readmission - even stronger than weight gain during hospitalization - for patients with severe AN. Taken together this research begins to piece together specific clinical markers that could be targeted to improve acute care.

Suicide is a leading cause of death for patients with EDs and a major public health problem worldwide. Additionally, active suicidality is one cause of emergency hospitalizations for patients with EDs (10). In this light, Zeppegno et al. reviewed the Interpersonal-Psychological Theory of Suicide across EDs aiming at disentangling the differences in individual suicidal behaviors. Three main constructs that need to be present in case of lethal suicide attempt were considered: Thwarted Belongingness, Perceived Burdensomeness, and Acquired Capability. After scrutiny of 10 studies also including patients undergoing high levels of care, Perceived Burdensomeness, namely the subjective experience of feeling themselves as a burden to their loved ones and self-directed anger and disgust, was found to be relevant with respect to the risk of suicidal ideation for patients with EDs.

This Research Topic was designed to promote a research debate around, and efforts to address, the complex needs of patients with acute and severe EDs receiving intensive therapeutic interventions. Relevant contributions were collected to expand knowledge on several aspects of full and partial hospitalization in EDs, across different ages, and encompassing predictors of outcome, efficacy of interventions, testing of novel therapeutic approaches, and follow-up outcomes. Nevertheless, in keeping with our initial aim to promote a scientific debate on these relevant matters, it is noted that future studies are warranted to investigate less well-known EDs (e.g., ARFID) and to test novel treatment strategies (e.g., innovative medications, online interventions, neuromodulation, combined approaches). The research literature on inpatients and partially hospitalized patients is challenging because studies involve patients with different demographic and clinical characteristics (e.g., BMI and weight trajectory) and with different levels of motivation for treatment. It is key to broaden the scope of this research to less studied and represented groups, such as those belonging to minority ethnic groups, non-binary gender, and people who live in marginalized geographical areas. Notwithstanding the need for further research, it is our opinion that the papers included in this Research Topic provide a valuable contribution to expanding knowledge on the very challenging topic of intensive treatment of patients with severe and enduring EDs. If much attention is being paid to improve early detection and treatment of these conditions, it is our hope that these efforts will be mirrored to improve outcomes and quality of life in those with longstanding difficulties.

## Author Contributions

EM wrote the first draft of the manuscript. RR, VC, and CL provided critical revision of the manuscript and important intellectual contributions. All authors read and approved the submitted version.

## Conflict of Interest

The authors declare that the research was conducted in the absence of any commercial or financial relationships that could be construed as a potential conflict of interest.

## Publisher's Note

All claims expressed in this article are solely those of the authors and do not necessarily represent those of their affiliated organizations, or those of the publisher, the editors and the reviewers. Any product that may be evaluated in this article, or claim that may be made by its manufacturer, is not guaranteed or endorsed by the publisher.
